# Book Reviews

**Published:** 1911-05

**Authors:** 


### BOOK REVIEWS

STATE BOARD QUESTIONS AND ANSWERS. By R. Max Goepp, M.D., Professor of Clinical Medicine at the Philadelphia Polyclinic. Second Edition Revised. Octavo volume of 715 pages. Philadelphia and London: W. B. Saunders & Company, 1911. Cloth, $4.00 net; Half Morocco, $5.50 net.

This book affords the applicant standing state board examinations a most valuable source of acquiring the information desired for these sometimes annoying examinations. It shows well the type of questions asked and gives a short but sufficient answer.

The reviewer knows of no book from which the student can better prepare himself for state board examinations.

---

A HANDBOOK OF PRACTICAL TREATMENT. In three volumes. By 79 eminent specialists. Edited by John H. Musser, M.D., Professor of Clinical Medicine, University of Pennsylvania; and A. O. Kelley, M.D., Assistant Professor of Medicine, University of Pennsylvania. Volume II; Octavo of 865 pages, illustrated. Philadelphia and London: W. B. Saunders Company, 1911. Per volume: Cloth, $6.00 net; half morocca, $7.50 net.

The second volume of this splendid work is well up to the standard of excellence set by the first volume. It begins with 152 pages on diseases of the cardiovascular system by Sir Clifford Allbutt, who is one of the most eminent of the profession in this branch of medicine. The simple, logical manner in which he discusses cardiac affections is worthy of the highest praise.

The various other diseases are ably discussed by such men as Dent, Cole, Finney, Hare, Weaver, Jopson, Otis, Pearce, Elting, White Wood, E. Martin, Shamberg, Barker, Carroll, Stengel, Bloodgood, McGlannan, Hamill, Chas. Martin, Dyer, Anderson, Rosenan, De Schweinitz, Richardson, Goldwait, and Reisman.

This work may be well taken as reflecting the best there is in modern medicine.

---

SURGICAL SUGGESTIONS. By Walter M. Brickner, M.D., and Eli Moschowitz, M.D. Surgery Publishing Co., New York.

This useful little volume might well be called the "active principles" of surgery, as it embodies many useful facts in very condensed form.

---

HANBOOK OF ELECTRO-THERAPEUTICS. By William J. Dugan, M.D., Lecturer on Electro-Therapeutics at Jefferson Medical College, Phila. 242 pages, with 91 Illustrations. Published by F. A. Davis & Co., Philadelphia.

This book differs widely from the majority of works on this subject, as it is much more condensed and practical. It is intended for physicians and students who know little or nothing of the subject. It is exact, clear and embraces the modern ideas of treatment by electrical measures.

---

SYMPTOMATIC AND REGIONAL THERAPEUTICS. By George H. Hoxie, A.M. ,M.D., Prof of Internal Medicine and Dean of the Clinical Department of the School of Medicine of the University of Kansas. 449 pages, with [illegible]8 illustrations. Publishers, D. Appleton & Co., New York.

This book represents the material collected for the course in general therapeutics recommended by the Committee on Currilum of the American Medical Association. The first part is devoted to a consideration of the symptoms and their relief; the second part to the therapy of inflammation. Finally, a review of the pathology of the body, region by region, is given and the principles already laid down applied to various disease-entities.

NEW AND NONOFFICIAL REMEDIES, 1911. Containing descriptions of articles which have been accepted by the Council on Pharmacy and Chemistry of the American Medical Association, prior to Jan. 1, 1911. Price, paper, 25c: Cloth, 50 cents. 282 pages.

This is the 1911 edition of the annual New and Nonofficial Remedies, issued by the Council on Pharmacy and Chemistry of the American Medical Association, and contains descriptions of all articles approved by the Council, up to Dec. 31, 1910. There are also descriptions of a number of unofficial non-proprietary articles which the Council deemed of value. The action, dosage, uses and tests of identity, purity and strength of articles are given.

In the arrangement and the scope of individual descriptions, the present edition does not differ widely from the 1910 edition, but it contains about twenty-five additional pages, these being required to describe the articles accepted by the Council during 1910.

